# Current Recommendations on Diagnosis and Management of Right-Sided Diverticulitis

**DOI:** 10.1155/2009/359485

**Published:** 2009-03-24

**Authors:** Dana A. Telem, Kerri E. Buch, Scott Q. Nguyen, Edward H. Chin, Kaare J. Weber, Celia M. Divino

**Affiliations:** Department of Surgery, The Mount Sinai School of Medicine, Box 1259, 1 Gustave L. Levy Place, New York, NY 10029, USA

## Abstract

We present the case of a 52-year-old female with recurrent symptomatic ascending colon diverticulitis who ultimately underwent elective laparoscopic right hemicolectomy. The following is a case report and literature review pertaining to right colonic diverticular disease.

## 1. Case Report

A 52-year-old female presented to the emergency department complaining of several years of right-sided abdominal pain which had recently become more acute and frequent. On presentation, she described right upper quadrant pain radiating to the right middle and lower quadrants. The pain was associated with nausea and decreased appetite, but she did not identify any exasperating events. She denied fevers, chills, chest pain, shortness of breath, or change in bowel function. In the preceding year, she had experienced multiple self-limited attacks of right upper quadrant abdominal pain. These symptoms prompted a workup which demonstrated cholelithiasis, and she underwent laparoscopic cholecystectomy. Initially her pain remitted, but returned several months later. Her past history was significant for gastroesophageal reflux disease, appendectomy ten years prior, cesarean section, abdominoplasty and right oophorectomy secondary to ovarian torsion.

On exam, the patient was afebrile and hemodynamically stable. Her abdominal exam was remarkable for moderate right upper, middle, and lower quadrant tenderness to palpation without guarding or rebound tenderness. Her bowel sounds were normoactive, and no hernias were appreciated. The remainder of her physical exam was unremarkable. Her leukocyte count was normal at 9.7 × 10^3^/mm^3^, as were the remainder of her laboratory values.

**Figure 1:**
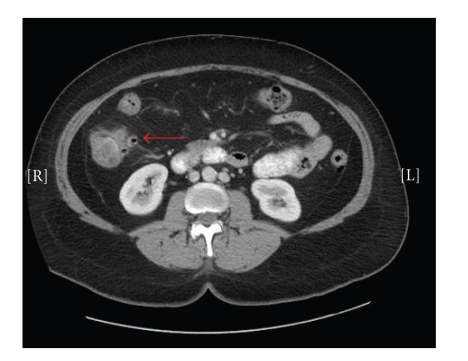
Transverse CT image demonstrating right-sided colonic diverticulitis.

**Figure 2:**
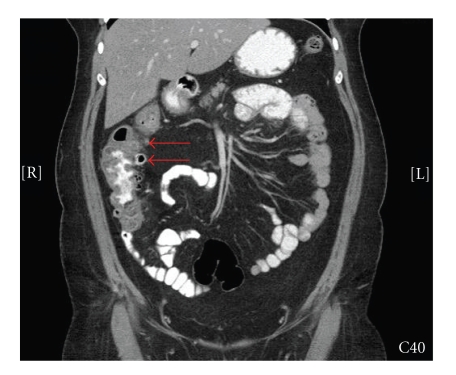
Coronal CT image demonstrating right-sided colonic diverticulitis.

A computed tomography (CT) scan was performed and demonstrated multiple ascending colon diverticula with pericolonic stranding and colonic wall thickening (Figures 1 and 2), consistent with right colonic diverticulitis. The patient was admitted to the hospital, placed on bowel rest, and started on intravenous antibiotic therapy. During the course of her hospitalization, her symptoms gradually resolved and she was discharged home on hospital day three. The patient was followed as an outpatient, at which time she elected for resection of the affected colon.

Six-weeks following her hospitalization, an uncomplicated laparoscopic right hemicolectomy with ileotransverse anastomosis was performed. Her postoperative course was unremarkable, and she was discharged on postoperative day four tolerating a regular diet. Specimen pathology revealed multiple ascending colonic diverticula. Currently, she remains asymptomatic with no recurrence of right-sided abdominal pain.

## 2. Literature Review

Diverticula are mucosal herniations that protrude through openings created by the vasa recta in the colon wall. In western countries, right-sided diverticulosis affects approximately 5% of the population and accounts for 1.5% of patients presenting with diverticulitis. Disease prevalence is significantly higher in Asian countries where right-sided diverticulosis accounts for 20% of patients with diverticular disease and 75% of cases of diverticulitis [1]. This discrepancy is assumed secondary to dietary and genetic factors. In comparison with patients of left-sided diverticular disease, patients with right colonic diverticular disease are younger at presentation, mean of 35 to 45 years of age, with equal gender distribution [2].

Right-sided diverticula may be solitary or numerous and can be found in the appendix, cecum, or throughout the ascending colon. When right-sided diverticula are solitary, they are usually congenital and true diverticula; when multiple, they are typically acquired and false diverticula. For acquired diverticula, increased intraluminal pressure and abnormal ascending colon motility play an important role in disease pathogenesis [3]. Patient presentation ranges from asymptomatic disease incidentally found on imaging study to gastrointestinal bleed or inflammatory process. Approximately 3% to 15% of patients with colonic diverticulosis will present with a gastrointestinal bleed. Bleeding frequently occurs at the neck of the diverticulum from the vasa recti. Though the majority of diverticular GI bleeds stop spontaneously, studies have demonstrated a significant recurrence rate quoted at 10% by 1 year and 50% by 10 years. Right-sided diverticula are responsible for greater than 50% of diverticular GI bleeds [4, 5].

When an inflammatory process occurs, right-sided diverticulitis often mimics appendicitis. Significant clinical findings suggestive of right-sided diverticulitis versus appendicitis include a low incidence of nausea, emesis, and anorexia accompanying the abdominal pain as well as variable point of maximum tenderness to palpation on abdominal exam [6]. Other etiologies which right-sided diverticulitis may mimic include cholecystitis, gastritis, and peptic ulcer disease [7]. Prior to routine use of radiographic imaging, the majority of patients were diagnosed at time of laparotomy. Several published series demonstrate that correct preoperative clinical diagnosis occurs in 4% to16% of cases. The reported incidence of finding diverticulitis during presumed appendectomy is quoted at 1 in 300 cases [6–8].

Diagnostic accuracy is essential, as the mainstay of therapy for right-colonic diverticulitis is medical rather than operative management. Historically, contrast enema was the procedure of choice for diagnosing right colonic diverticula. Though accurate, this technique is limited to asymptomatic patients secondary to risk of perforation during an acute disease flare. Currently, CT scan, ultrasound (US), and magnetic resonance (MR) imaging have all been described as effective modalities to preoperatively differentiate right-sided diverticulitis from other intra-abdominal pathology.

CT scan in many institutions has become the diagnostic modality of choice to delineate the etiology of right-sided abdominal pain. Findings on CT scan consistent with a diagnosis of right colon diverticulitis are similar to those appreciated with left-sided disease. Findings include colonic wall thickening, presence of extraluminal mass, haziness and stranding of adjacent pericolic fat, and thickening of nearby fascial planes [9–12]. Though CT scan has a documented diagnostic accuracy rate of 90% to 95%, right-sided diverticulitis may still be radiographically mistaken for appendicitis with abscess, Crohn's disease, omental infarction, or colon cancer [9–11].

Another widely used modality for assessing right-sided abdominal pain is US. US confers many advantages over CT scan as it does not use ionizing radiation, is readily available in almost every hospital, and is cost effective. The use of US in diagnosing right-sided diverticulitis has been heavily investigated. On US, the presence of rounded hypo- or anechoic structures that protrude from thickened bowel wall, with or without strong echoes representing gas, feces or stone, is consistent with a diagnosis of right-colonic diverticulitis [13]. US for right-sided diverticulitis, when performed by an experienced operator, has quoted 91.3% sensitivity and 99.8% specificity for correct diagnosis [9, 12–14].

Though CT and US both have a high sensitivity and specificity for diagnosing right colon diverticulitis, they have limitations. US is variable and operator dependent; several reports describe cases of right colonic diverticulitis being misdiagnosed as appendicitis with fecalith resulting in unnecessary operative intervention [15]. CT scans utilize ionizing radiation which is a relative contraindication in pregnancy and for young patients. For these reasons, MR has also been explored as a diagnostic option. A recent study from the Netherlands demonstrated MR to accurately diagnose patients with right colonic diverticulitis [16]. Though availability and use of MR is limited in some hospitals, it may be a valuable alternative in select patients with contraindications to CT scan in whom US is nondiagnostic.

The treatment of right-sided diverticula depends on severity of presentation and modality of diagnosis. Asymptomatic diverticula incidentally found on imaging do not require intervention. Diverticula presenting as a GI bleed are initially managed conservatively with hemodynamic support as 75% of episodes are self-limited. If bleeding persists, endoscopic intervention should be attempted. In cases where endoscopic management fails, right hemicolectomy may be necessary [4, 5]. For patients with recurrent GI bleed from right colon diverticula requiring multiple transfusions or hospitalizations, the authors recommend consideration of elective right hemicolectomy.

With the exception of isolated cecal diverticulitis, no consensus currently exists on optimal treatment of patients with right-sided colonic diverticulitis found incidentally at time of operation. While some surgeons advocate no intervention, others recommend at minimum appendectomy or diverticulectomy if inflammation is minimal. Right hemicolectomy is reserved for extensive inflammation, perforation, or mass suspicious for carcinoma [17]. In cases of isolated cecal diverticulitis, resection is strongly recommended [18].

If a preoperative diagnosis of uncomplicated diverticulitis is made, patient management should consist of bowel rest and intravenous antibiotics. Right-sided diverticulitis differs from left-colon diverticulitis as it has a more indolent course. Several published series demonstrate long-term remission and control of disease solely with medical therapy. Komuta et al. published a study demonstrating 99% of patients preoperatively diagnosed with uncomplicated right colon diverticulitis were successfully treated with bowel rest and antibiotics. Over an average of 3 years, 20% experienced a recurrent attack of uncomplicated diverticulitis all of whom resolved with medical therapy. Of the 20% who recurred, 15% experienced a third attack. Again, all patients who recurred a third time had uncomplicated presentations and were successfully treated without operative intervention [19]. Another recently published study examined the management and outcome of 113 patients with right colon diverticulitis over 10 years. This paper again demonstrated an uncomplicated recurrence rate of 20% [20]. In contrast to recommendations for left-colon disease, age and frequency of attacks should not prompt elective colon resection as recurrence requiring emergent intervention is rare [21]. Elective resection, however, should be considered in cases of frequent recurrence that interfere with activities of daily living as was the case in our patient.

An exception to continued medical therapy is isolated cecal diverticulitis. Cecal diverticulitis is an uncommon occurrence which is rarely preoperatively diagnosed. Surgical therapy ranges from diverticulectomy with or without cecectomy to right hemicolectomy depending on the extent of inflammation. Most surgeons advocate aggressive resection, as cecal diverticulitis infrequently resolves with medical therapy and has a high rate of complicated recurrence [22, 23].

For patients presenting with complicated right colon diverticulitis, initial therapy is similar to patients with left colon diverticulitis. Patients who present with abscess, but are otherwise hemodynamically stable, should be treated with percutaneous abscess drainage, bowel rest, and intravenous antibiotics. Though uncommon, patients with overt perforation or who are clinically unstable should be taken for immediate operative intervention.

## 3. Conclusion

Right colon diverticulitis is a rare entity in the West which is frequently mistaken for other diseases processes, most commonly appendicitis. Radiographic imaging, with either CT scan or US, is essential for proper diagnosis as the mainstay of therapy is medical rather than operative management. Though imaging has greatly decreased unnecessary operative intervention; right colonic diverticulitis is still incidentally encountered at time of operation and treatment should be tailored to the extent of disease process. In cases of incidental operative discovery where a normal appendix is found and colonic inflammation minimal, our recommendation is that no intervention be undertaken. For cases of uncomplicated diverticulitis accurately diagnosed prior to operative intervention, initial therapy should consist of bowel rest with intravenous antibiotics, even in cases of recurrence. Elective resection should be considered based on patient preference or in cases where malignancy is suspected. Complicated diverticulitis presenting as abscess should be treated either by percutaneous abscess drainage or by operative intervention in cases of patient instability. Patients who present with hemodynamic instability or perforation should undergo emergent operative intervention.
